# Procalcitonin in Medullary Thyroid Carcinoma: An Umbrella Review of Systematic Reviews and Meta-Analyses

**DOI:** 10.3390/jcm15218255

**Published:** 2026-10-23

**Authors:** Petra Petranović Ovčariček, Alfredo Campennì, Rosaria Maddalena Ruggeri, Marija Punda, Andrija Gregov, Ana Bejuk, Alberto F. Campennì, Martin W. Huellner, Luca Giovanella

**Affiliations:** 1 School of Medicine, University of Zagreb, Šalata 3, 10000 Zagreb, Croatia; 2 Department of Oncology and Nuclear Medicine, University Hospital Center Sestre Milosrdnice, Vinogradska 29, 10000 Zagreb, Croatia; 3 Department of Biomedical and Dental Sciences and Morpho-Functional Imaging, University of Messina, 98100 Messina, Italy; acampenni@unime.it; 4 Endocrinology Unit, Department of Human Pathology of Adulthood and Childhood DETEV, 98125 Messina, Italy; rmruggeri@unime.it; 5 Department of Radiological Technology, University of Applied Health Sciences, Mlinarska Cesta 38, 10000 Zagreb, Croatia; 6 Medicine and Surgery Bachelor’s Degree Course, Department of Human Pathology of Adulthood and Childhood DETEV “G. Barresi”, University of Messina, 98125 Messina, Italy; albertocampenni@icloud.com; 7 Department of Nuclear Medicine, University Hospital Zürich, Rämistrasse 100, 8091 Zürich, Switzerland; 8 Department of Nuclear Medicine, University of Zürich, Pestalozzistrasse 3, 8032 Zürich, Switzerland; 9 Department of Nuclear Medicine and Thyroid Center, Gruppo Ospedaliero Moncucco, Via Soldino 5, 6900 Lugano, Switzerland

**Keywords:** calcitonin, evidence synthesis, medullary thyroid carcinoma, procalcitonin, systematic review, tumour marker

## Abstract

**Background/Objectives:** Procalcitonin has been proposed as an alternative or complementary biomarker to calcitonin in medullary thyroid carcinoma (MTC). We synthesised the published systematic reviews, appraised their quality, and measured their reliance on the same primary studies. **Methods:** Umbrella review of systematic reviews reporting measured procalcitonin values in proven or suspected MTC. PubMed/MEDLINE and the Cochrane Library were searched to 30 June 2026. Quality was appraised with AMSTAR-2 and overlap as the corrected covered area (CCA). **Results:** Seven evidence syntheses (2015–2024) were included, four addressing procalcitonin itself and two with meta-analysis. Across diagnostic and monitoring populations, a bivariate meta-analysis of 11 studies and 5817 patients gave sensitivity of 0.90 (95% CI 0.71–0.97) and specificity of 1.00 (95% CI 0.85–1.00); in postoperative surveillance, five studies and 296 patients gave pooled sensitivity and specificity of 96%. The five syntheses enumerating their sources drew on 21 distinct primary reports, giving a corrected covered area of 38.1%, every pairwise value above the 15% threshold for very high overlap. Two primary studies were extracted discordantly; for one of them the same data yielded specificities of 84% and 5% depending on the threshold and assay platform applied. Confidence was low for one review and critically low for six. **Conclusions:** Procalcitonin is a promising adjunct to calcitonin, with better analytical behaviour, but routine combined measurement is not yet supported. The apparent consistency across syntheses reflects a shared and narrow primary literature rather than independent replication, and the absence of an agreed, method-specific threshold remains the main barrier to implementation.

## 1. Introduction

Calcitonin has been the reference biomarker of MTC for half a century, and nothing currently available displaces it [1,2,3,4,5,6]. However, the analyte is degraded by serum proteases at room temperature and loses roughly a quarter of its concentration within twelve hours even under refrigeration, so samples must be separated and chilled promptly [7,8,9]. Detected immunoreactive isoforms and fragments differ between methods, and inter-assay and inter-laboratory agreement is correspondingly poor [10]. Heterophile antibodies, macro-analyte formation and very high tumour burden may all produce misleading results [11,12,13,14]. Biological confounders compound the analytical ones. Renal impairment, hyperparathyroidism, neuroendocrine neoplasia at other sites, proton pump inhibitor use, smoking and thyroid volume itself all raise circulating calcitonin, so a mildly elevated result in a patient with a thyroid nodule poses a problem rather than answering one [1,15,16].

Procalcitonin, the 116-amino-acid precursor from which calcitonin is cleaved [17,18], has been proposed as a way past several of these difficulties [19,20,21]. It circulates with a concentration-independent half-life of 20 to 24 h, it withstands ordinary transport and storage without a cold chain, and the intellectual property underlying commercial assays derives from a single licensor, so that methods in routine use are closely comparable [22,23]. Those properties are attractive to any laboratory that has tried to reconcile calcitonin results across platforms.

The proposition has been examined repeatedly. Two systematic reviews appeared in 2015, one devoted to procalcitonin itself [24] and one to MTC without calcitonin secretion [25], and a third followed in 2016 [26]. A meta-analysis of postoperative performance was published in 2018 [27], and a bivariate meta-analysis covering both diagnosis and monitoring in 2021 [28]. Broader syntheses of circulating markers in MTC have since incorporated procalcitonin among several candidates, and a comparative meta-analysis of pro-gastrin-releasing peptide places it alongside a further contender [29].

The syntheses were built on different questions, different eligibility rules and different reference standards, and above all on different assumptions about what constitutes a positive result. They also draw on a small primary literature, so their apparent concordance may reflect common sources rather than independent replication.

We therefore conducted an umbrella review. An earlier umbrella review addressed the full range of different diagnostic tests in MTC, treating procalcitonin as one item among many [30]; the present work is specifically focused on procalcitonin, extends the search by more than three years, adds a formal analysis of overlap, and is framed around the analytical and pre-analytical considerations that determine whether a marker can be implemented in routine practice.

## 2. Materials and Methods

### 2.1. Design, Protocol and Reporting

This is a review of systematic reviews. It was conducted following the methodological guidance of Aromataris and colleagues [31] and reported in accordance with the PRISMA 2020 statement [32] and, where applicable, with the PRIOR reporting guideline for overviews of reviews [33]. The protocol was agreed among the authors before the search was run and was registered retrospectively at the Open Science Framework (DOI: 10.17605/OSF.IO/B97FK) after completion of data extraction and before submission; the retrospective timing is stated explicitly because registration counts as a critical domain of the appraisal instrument we applied to others.

### 2.2. Eligibility Criteria

Systematic reviews, with or without meta-analysis, were eligible if they reported measured values of serum or plasma procalcitonin in the diagnosis or the follow-up of MTC, in any clinical setting and at any point in the care pathway. Eligible reports also had to describe a reproducible search of at least one bibliographic database together with explicit selection criteria. Narrative reviews, primary studies, case reports, editorials and conference abstracts were excluded. No language restriction was applied and no earliest publication date was set; both databases were searched from inception to the date the search was run.

### 2.3. Search and Study Selection

PubMed/MEDLINE and the Cochrane Library were searched from inception to 30 June 2026, combining terms for medullary thyroid carcinoma with terms for procalcitonin and with the systematic review and meta-analysis publication types. Reference lists of the retrieved reports and of the earlier umbrella review [30] were screened. Two reviewers screened titles and abstracts independently and examined the full text of every record retained, with disagreement resolved by discussion. The flow of records through identification, screening and inclusion is shown in Figure S1, the full search strategies in Supplementary Table S1, and the completed PRISMA 2020 checklist is provided as Supplementary Material.

Extraction was performed in duplicate on a piloted form, against the full text in every case.

### 2.4. Methodological Quality

Quality was appraised with AMSTAR-2 [34]. Overall confidence was classified as high, moderate, low or critically low according to the number of critical domains judged deficient, the critical domains being items 2, 4, 7, 9, 11, 13 and 15. AMSTAR-2 was developed for reviews of interventions and its application to reviews of diagnostic accuracy requires interpretation, particularly of items 9 and 11, where QUADAS-2 [35] and hierarchical modelling respectively are the appropriate standards. We therefore required a hierarchical (bivariate or HSROC) model for a yes on item 11, separate univariate pooling of sensitivity and specificity being judged inappropriate, and accepted Deeks’ funnel plot asymmetry test as the appropriate investigation of publication bias under item 15. No other item was modified. The response options were those of the original instrument, partial yes being used only for items 2, 4, 7, 8 and 9, where the instrument provides it. We report the domain judgements alongside the summary rating so that readers can weigh them [36].

### 2.5. Overlap of Primary Studies

Double counting, by which we mean the inclusion of one primary study in more than one synthesis, is expected in a field this size and is not in itself a fault; it matters only because it inflates the apparent weight of evidence. We therefore constructed a citation matrix of every primary report on procalcitonin included in each different synthesis, and computed the corrected covered area (CCA) by the method of Pieper and colleagues [37], as (N—r) divided by (rc—r), where N is the number of filled cells, r the number of distinct primary reports and c the number of syntheses. Because the syntheses span seven years, some cells could not have been filled: a study published after a review’s search date could not have been included, so its absence carries no information about that review’s selection and is treated as a structural zero. We therefore repeated the calculation with structural zeros removed from the denominator, and we also computed CCA for every pair of syntheses. The review of calcitonin-negative disease was not represented in the matrix, since its eligibility was not defined by procalcitonin. Where the same primary data appeared in more than one synthesis, we compared the accuracy figures attributed to it.

### 2.6. Synthesis

Pooling the syntheses themselves would have counted the same primary studies more than once, and was therefore not attempted. Accordingly, findings were synthesised narratively and organised by clinical setting. Where two syntheses reported discrepant values for the same underlying data, both are given, and the source of the discrepancy is identified.

### 2.7. Certainty of Evidence

Confidence in a review, as rated by AMSTAR-2, is not the same as certainty in a clinical finding, and the two were assessed separately. Certainty in each evidence statement was judged with the GRADE approach for test accuracy [38,39]. Ratings were assigned by P.P.O. and L.G. and agreed by discussion. Evidence from cross-sectional or cohort accuracy studies started as high certainty and was rated down for risk of bias, including incorporation of calcitonin into the reference standard and thresholds derived on the data in which they were then tested; for indirectness, where the populations pooled did not match the clinical question; for inconsistency; for imprecision; and for suspected publication bias. Agreement between syntheses that shared primary studies was not counted as independent confirmation. Statements resting on a single cohort or on case-level data were rated low or very low. Statements describing the state of the literature rather than an accuracy estimate, such as the absence of a validated threshold, were rated on the consistency and completeness of the evidence behind them. The reasons for rating down are explained below. The registered protocol specified a certainty judgement for each statement based on the number and size of contributing studies, consistency, overlap and risk of bias; the GRADE approach was adopted during peer review to make these criteria explicit, together with the specification of Deeks’ test under AMSTAR-2 item 15, and both are reported here as amendments to the protocol.

## 3. Results

### 3.1. Included Evidence Syntheses

Seven evidence syntheses published between 2015 and 2024 met the inclusion criteria [24,25,26,27,28,40,41]. Four addressed procalcitonin in MTC as the primary question [24,26,27,28], two examined MTC without calcitonin secretion and between them reported procalcitonin in three of 119 patients [25,41], and one surveyed circulating markers in MTC generally [40]. Two incorporated meta-analysis [27,28]. Their characteristics are set out in Table 1. Two further evidence syntheses were assessed in full text and excluded because neither reported a measured procalcitonin value. The first, a 2025 comparative bivariate meta-analysis of pro-gastrin-releasing peptide, evaluates a different index test and reports no procalcitonin accuracy data; it is considered in the Discussion as comparator evidence [29]. The second, a 2019 systematic review of MTC without calcitonin secretion [42], assembled 49 patients and named procalcitonin among the markers that should be sought in this phenotype, but measured it in none of them. The two 2015 reviews [24,25] and the 2016 review were qualitative [26]; the first quantitative synthesis appeared in 2018 [27] and was confined to the postoperative setting; the first to apply bivariate methods and to address the preoperative question appeared in 2021 [28], the broad marker review in 2022 [40] and the larger of the two syntheses of calcitonin-negative disease, which contributes two of the three measured procalcitonin values, in 2024 [41].

### 3.2. Methodological Quality

Overall confidence was critically low for six of the seven syntheses and low for the remaining one (Table 2). Two problems recur. First, only one review reported a registered protocol [28] before the search was run and, second, only one named the reports excluded at full-text stage together with the reason for each. Both are critical domains, so a single deficiency lowers the rating irrespective of performance elsewhere. Three reviews restricted eligibility by language [26,40,41] and one by publication date [40], which is enough on its own to fail the search domain: AMSTAR-2 allows a partial yes on item 4 only where publication restrictions are justified, and none of the three gave a reason.

The domain judgements are given in Table 2. The 2018 meta-analysis applied QUADAS-2, quantified heterogeneity and tested for publication bias [27]; it also names each report excluded after full-text assessment together with the reason, but it pooled sensitivity and specificity separately with univariate random-effects models, which ignores the correlation between them, and it had no registered protocol, a convention not yet standard at the time. With two critical deficiencies its overall confidence is critically low. Its Egger test, moreover, is not the test recommended for accuracy data and has little power with five studies. The 2021 meta-analysis was registered in PROSPERO before commencement, applied bivariate and hierarchical models appropriate to diagnostic accuracy data rather than pooling sensitivity and specificity independently, assessed publication bias by Deeks’ test and carried out predefined subgroup analyses and meta-regression [28]. Its only critical deficiency is the absence of a list of the reports excluded at full-text stage, the flow diagram giving the reasons in aggregate; it is the only synthesis rated low rather than critically low. At the other end, no pooled data were provided by the review of calcitonin-negative disease and the broad marker review [25,40]. The 2015 review of the non-secretory phenotype searched three databases and discussed analytical interference at length but appraised none of its sources formally [25]. It is, however, a useful inventory of a rare presentation and should be read as such.

### 3.3. Overlap of the Primary Evidence Base

The five syntheses that enumerated their procalcitonin sources made between them 53 study inclusions drawn from 21 distinct primary reports (Table 3). Only these five syntheses enter the calculation, and its unit is the publication, not the independent patient cohort. The corrected covered area is 38.1%, against a threshold of 15% conventionally taken to denote very high overlap [37]. Removing structural zeros, that is, cells that could not have been filled because the primary study postdated the review’s search, raises the value to 44.4%. Restricting the comparison to the studies actually pooled gives 23.1% between the five studies of the 2018 meta-analysis and the eleven of the 2021 meta-analysis, which share three. The distinct reports are fewer still when considered as independent datasets. Two of them share a cohort of disease-free patients [43,44]; both were included by one synthesis [24] and excluded on that ground by another [27]. The conference abstract of Lim and colleagues [45] and their later full paper [46] report the same 476 samples and the same predictive values, and originated from the same laboratory. Six of the 21 reports are single case descriptions [47,48,49] or unpublished conference material [49,50,51], and one of the eleven pooled in the 2021 meta-analysis was a conference abstract [50].

The concordance between the 2018 and 2021 estimates should therefore not be read as replication. The two analyses share primary data, authors, assays and reference standard, so their agreement shows internal consistency but says little about generalisability. This does not make the confidence interval of either meta-analysis incorrect; it means that the second adds little independent confirmation to the first.

### 3.4. Discordant Extraction of the Same Primary Data

Two primary studies were extracted differently by the syntheses that shared them. The series of Algeciras-Schimnich and colleagues [54] appears in all five reviews that enumerate their sources. One extracted 133 patients, classified 91 as having active disease and derived a sensitivity of 91.2% with a specificity of 100% [24]. Another extracted the same 133 patients, restricted the target condition to the 43 with proven structural recurrence, and derived a sensitivity of 97.7% [27]. A third extracted 590 patients from the same report, of whom 83 were true positives, 26 false positives, eight false negatives, and 473 true negatives, giving a sensitivity of 91.2% and a specificity of 94.8% [28]. A fourth reported a sensitivity of 91.2% with a fraction attached to it that does not evaluate to that percentage [40]. A fifth reproduced three further pairs according to the comparison group taken, 91% and 99.5% against healthy controls, 98% and 100% against other thyroid diseases, and 96% and 99% against all other cases [26]. Five renderings, three patient denominators, and six distinct pairs of sensitivity and specificity were produced by the same primary study.

The series of Walter and colleagues [56] shows the same effect more starkly, and here the cause is explicit. One review applied a threshold of 0.5 μg/L that the primary authors had derived for discriminating MTC from healthy controls, and reported sensitivity and specificity of 84% each [24]. A second applied a threshold of 0.06 μg/L and reported a sensitivity of 97.1%, with specificity not calculable [27]. A third applied 0.15 μg/L and, from 67 true positives, 40 false positives, two false negatives and two true negatives, obtained a specificity of approximately 5%, with 40 of its 42 comparison patients falling above that value [28]. The collapse is method-dependent rather than biological: that series was measured on a chemiluminescence platform rather than the time-resolved amplified cryptate emission (TRACE) method used by most of the pooled studies, and its own authors derived 0.5 μg/L as the value discriminating MTC from their comparison groups, so imposing a threshold of 0.15 μg/L drawn from other platforms places almost every comparator above it. That study was one of two identified as statistical outliers in the bivariate analysis, and its exclusion in sensitivity analysis left the summary estimates essentially unchanged apart from a fall in the area under the curve from 0.97 to 0.90 [28]. Neither discrepancy is an error. The relevant point here is that the extracted figures depend on which patient groups and which threshold were taken, so accuracy estimates for procalcitonin in MTC are uninformative without specifying them. The opposite pattern is also present, and the citation matrix does not capture it. The 2022 review reports seven postoperative sensitivities and five specificities for procalcitonin. Six of those sensitivities and four of the specificities are numerically identical to the corresponding entries in the 2015 systematic review and appear in the same order of studies; the remaining two are drawn from the 2018 meta-analysis [24,40]. Two of those figures are specific to the 2015 review rather than to the primary papers: 84% for the series of Walter and colleagues [56], which follows from the threshold that review chose rather than from the primary paper, and 57.1% for the series of Bolko and colleagues [53], which is that review’s own tabulation of eight of 14 cured patients. The 2015 review is cited within the same section of the 2022 paper. Whatever the route, these are not two independent extractions of the primary literature, and the apparent accumulation of confirmatory syntheses is smaller than the count of syntheses suggests.

### 3.5. Procalcitonin in the Preoperative Setting

One synthesis pooled diagnostic performance across eleven studies [28]. Its authors describe the population as patients with nodular thyroid disease, but four of the eleven studies, with 364 patients, were exclusively postoperative and at least two others combined postoperative patients with newly diagnosed patients or controls, so the pooled estimate mixes diagnostic and monitoring data and is not a purely preoperative one. Across the 5817 patients, of whom 336 had MTC according to the text of that report (its forest plot lists 391 reference-positive units), summary sensitivity was 0.90 (95% CI 0.71–0.97) and summary specificity 1.00 (95% CI 0.85–1.00) for thresholds between 0.06 and 0.50 μg/L. The positive likelihood ratio was 288 (95% CI 5.6–14,929.3), the negative likelihood ratio 0.10 (95% CI 0.03–0.33), and the area under the curve 0.97 (95% CI 0.95–0.98). Excluding the two outlying studies left nine studies, 5654 patients and a prevalence of 4.6%, with sensitivity of 0.90 (95% CI 0.85–0.93), specificity of 1.00 (95% CI 0.96–1.00) and a lower area under the curve of 0.90 (95% CI 0.87–0.93).

Meta-regression in that review identified the assay platform as a significant influence on both sensitivity and specificity, and the threshold as a significant influence on specificity [28]. Seven of the eleven studies used TRACE and four used other platforms; the pooled diagnostic odds ratios were of similar magnitude, but the heterogeneity differed appreciably. This is direct evidence that the accuracy of procalcitonin for MTC is method-dependent, and it has not been carried into any current recommendation.

The two earlier qualitative syntheses reached comparable conclusions without pooling data. The small 2015 review [24] examined two preoperative studies. In the larger, 107 of 112 patients in whom both markers were measured before surgery had procalcitonin above 0.1 μg/L, a sensitivity of 95.5% [59]. In the second, 1236 patients with thyroid nodules yielded 14 with calcitonin above 10 ng/L, of whom two had MTC; basal and stimulated procalcitonin were raised in those two patients alone, giving positive and negative predictive values of 100% in that series [58]. The 2016 review examined fifteen reports and concluded that procalcitonin belonged alongside calcitonin rather than in its place [26].

### 3.6. Procalcitonin in Postoperative Surveillance

The 2018 meta-analysis identified eight reports of procalcitonin during follow-up after treatment for MTC and pooled five of them, comprising 296 patients, of whom 140 had proven recurrence [27]. Pooled sensitivity for the detection of recurrent or persistent disease was 96% (95% CI 92–99), with an I-squared of 0% whose confidence interval (0–64.1%) does not exclude substantial heterogeneity, and a non-significant Egger test (3.16, *p* = 0.99), which with five studies cannot be taken to show that publication bias is absent. Pooled specificity was 96% (95% CI 87–100) but rested on three of the five studies, since two reported no patients classified as free of disease, and heterogeneity was appreciable (I-squared 66.6%). The 2021 meta-analysis reported a postoperative subgroup of four studies and 364 patients, in whom MTC prevalence was 48.6%, with sensitivity of 0.93 (95% CI 0.85–0.97) and specificity of 0.91 (95% CI 0.20–1.00); the last confidence interval is so wide as to be uninformative [28].

The 2015 systematic review reported the same setting without pooling, on seven studies and 265 patients, of whom 114 were cured and 151 had recurrent or persistent disease [24]. Sensitivity in recurrent disease was 100% in four series and 91.2% in the largest; specificity in cured patients was 100% in one series, 87.0% in two, and 57.1% in the earliest, which measured procalcitonin with an immunochromatographic kit and treated values above 0.5 μg/L as positive, a threshold well above that used in the others [53]. That spread of specificity, from 57% to 100%, is a fair representation of what the primary literature actually contains, and it is not visible in either pooled estimate.

The most substantial primary contribution comes from the series described above in which procalcitonin above 0.15 μg/L identified 42 of 43 patients with structural recurrence and remained below that value in all 42 patients free of disease [54]. In the subgroup of 91 patients from that series in whom both markers were measured, calcitonin and procalcitonin, each identified 83 and each missed eight; seven of the eight missed by one marker were detected by the other, and only one patient was missed by both, so the two markers used together reached a sensitivity of 98.9%. That figure is the single strongest argument in the literature for measuring the two markers in parallel rather than choosing between them, and it rests on one cohort.

### 3.7. Calcitonin-Negative Medullary Thyroid Carcinoma

The clinical situation in which an alternative marker would be most valuable is the one in which the evidence is thinnest. The 2015 systematic review of MTC without calcitonin secretion assembled eleven reports describing eighteen histologically proven cases [25]. Calcitonin was below the institutional cut-off in every case and undetectable in four. Carcinoembryonic antigen was measured in eleven patients and was normal in ten; chromogranin A was reported as measured in two patients and normal in both. Procalcitonin was measured in one patient, falling from 0.21 μg/L before surgery to below 0.1 μg/L afterwards. A second systematic review, published in 2024 [41], assembled 101 patients from 32 reports and measured procalcitonin in only two of them, raised before surgery and normal afterwards in one and normal throughout in the other; a third patient in that series had a relapse identified by procalcitonin when every other neuroendocrine marker was negative. A third review, published in 2019, extended the earlier series to nineteen reports and forty-nine patients and reported no procalcitonin value for any of them; it is excluded from this review on that ground [42]. Three measurements across 101 patients, in the largest series assembled [41], are not an evidence base. Both reviews set out the condition on which the proposal rests, and it is a conditional one: if the phenotype arises from aberrant processing of mature calcitonin, procalcitonin should be raised; if it arises from a defect of the secretory apparatus, procalcitonin should be low as well [25]. The same conditional is stated in the 2024 review, which does not cite the 2015 one and traces the point to a case report that cites it elsewhere but not for this mechanism, so the framing has been arrived at more than once even though no group has tested it. Both mechanisms have been proposed and neither has been excluded [41]. This should be stated whenever procalcitonin is recommended for this indication.

### 3.8. Decision Thresholds

Every synthesis that addressed the question reached the same conclusion, and none resolved it. The 2015 review tabulated the thresholds in use as 0.1, 0.15, 0.25 and 0.5 μg/L across eight studies and noted that concentrations up to 0.28 μg/L had been recorded in healthy subjects and up to 0.60 μg/L in athyreotic patients, which straddles all four of those values [24]. The 2016 review recorded that the data were insufficient to recommend a threshold while observing that most studies had settled on 0.1 μg/L [26]. The 2018 meta-analysis pooled studies applying 0.06, 0.15 and 0.32 μg/L, two of them derived by receiver operating characteristic analysis on the same data in which they were validated, and named this as a source of bias affecting both pooled estimates [27]. The 2021 meta-analysis pooled thresholds from 0.06 to 0.50 μg/L, found by meta-regression that the threshold significantly influenced specificity, and reported that the subgroup using values above 0.15 μg/L had a lower sensitivity of 0.86 (95% CI 0.64–0.96) [28]. The earlier umbrella review of MTC diagnostic tests reached the same position in 2023 [30]. An eightfold range of decision limits, several of them optimistically derived and one of them demonstrably shifting the pooled specificity, is not a detail.

### 3.9. Comparative and Complementary Markers

Ten of the 76 reports retained by the broad review of circulating markers addressed procalcitonin, and its extracted figures fall into the two settings already described: preoperative sensitivity of 95.5% in the largest series, and postoperative sensitivity of 84% to 100% with specificity of 57.1% to 100% [40]. The broad review did not compare the markers directly. The 2025 meta-analysis of pro-gastrin-releasing peptide, considered in the Discussion, adds a point that has not been examined for procalcitonin: the sensitivity of that marker was strongly size-dependent, reaching 100% at 50 pg/mL for tumours of 10 mm or more but falling to 24% or less below that size, with pro-gastrin-releasing peptide rising as the 1.23rd power of tumour diameter [29]. Whether procalcitonin behaves the same way at the thresholds in current use is unknown, because no synthesis has stratified it by tumour size.

Prognostic performance is a different question from diagnostic accuracy, and no included synthesis addressed it for procalcitonin. Doubling times of calcitonin and carcinoembryonic antigen are established prognostic factors [65,66,67], and carcinoembryonic antigen doubling time discriminates high-risk patients better than calcitonin doubling time does [65]. The first direct comparison of procalcitonin kinetics with calcitonin kinetics appeared very recently, after the last of the included syntheses. In 25 patients followed for up to 24 months after thyroidectomy, with both analytes measured by electrochemiluminescence on a single platform, doubling times derived from rolling three-point log-linear regression correlated closely (Spearman rho 0.91, 95% CI 0.80–0.96), showed a bias ratio of 1.04 with limits of agreement from 0.78 to 1.32, and agreed substantially across the clinically relevant strata of under 6, 6 to 12, 12 to 24 and over 24 months (Cohen kappa 0.76, 95% CI 0.65–0.95) [68]. Procalcitonin was undetectable at every time point in all fourteen patients with an excellent response. The cohort is small, and the authors present the work as exploratory, but the gap identified by every previous synthesis has begun to close. Agreement between two kinetic indices is not, however, evidence of prognostic value, which requires that procalcitonin doubling time be related to outcome. The evidence statements that the seven syntheses support, with the certainty attached to each, are summarised in Table 4.

## 4. Discussion

Three conclusions follow from this appraisal. Procalcitonin appears to perform well in both the preoperative and the postoperative setting, on estimates that are internally consistent. Those estimates rest on a primary literature so small and so extensively shared, with a corrected covered area of 38% overall and up to 73% between individual pairs of reviews, and with at least one review reproducing another’s extraction, figure for figure, that their agreement cannot be read as independent confirmation, even though the confidence interval within each meta-analysis remains valid on its own terms. The single question that would allow the marker to be implemented, namely which concentration should trigger which action on which platform, has been asked in every synthesis for eleven years, and no externally validated answer has emerged. Importantly, this uncertainty does not merely reflect a lack of clinical validation but also an unresolved and more fundamental problem in laboratory medicine: currently available immunoassays are sufficiently harmonised to permit universal, method-independent decision thresholds. This limitation is by no means unique to procalcitonin and affects, to varying degrees, most circulating tumour markers, and calcitonin even more so, as discussed below.

The reference standard is part of the problem. The strongest of the included syntheses states the difficulty plainly, and the recent meta-analysis of pro-gastrin-releasing peptide records it independently [29]. Eligible studies were those comparing procalcitonin with calcitonin, with calcitonin incorporated into the reference standard used to define cases. In clinical practice, an elevated calcitonin level typically triggers the diagnostic work-up that ultimately establishes the diagnosis [70,71,72]. Thus, calcitonin forms part of the reference standard against which its proposed alternative is evaluated, introducing an inherent degree of incorporation bias. The 2025 synthesis reaches the same conclusion independently for a different marker [29]. The corollary is less often drawn. False negatives of procalcitonin are ascertainable in this design, because calcitonin identified the case; false negatives of calcitonin are largely invisible, because nothing prompted the work-up. Comparative accuracy figures derived this way are systematically unfavourable to the new marker and systematically flattering to the old one, and the estimates in Table 1 should be read in that light.

Another consequence stems from the way the primary series were designed. The largest of these studies enrolled several distinct patient groups within a single cohort, including patients with neuroendocrine tumours of non-thyroid origin, newly diagnosed MTC, MTC in remission or with structural relapse, as well as patients with non-medullary thyroid disease. Each synthesis extracted from that report the subgroups that matched the question it was asking, which is why the same series yields a different denominator and a different pair of estimates in each of them, and why those figures are not interchangeable. For the diagnostic question in particular, the only admissible values are preoperative concentrations in patients whose disease was subsequently confirmed histologically; several of the pooled estimates rest in part on postoperative surveillance data or on comparison groups assembled for a different purpose, and are correspondingly optimistic. Histology, cytology with calcitonin immunocytochemistry, or calcitonin measured in fine-needle aspirate washout (although it also has false-positive results) would each serve; the 2021 authors propose exactly this in their closing paragraph [28]. For procalcitonin, one such study has since been published, a prospective single-centre series of 478 patients undergoing thyroidectomy for nodular disease, 23 of them with MTC, in which both markers were measured before surgery and verified histologically [73]. Calcitonin was the more sensitive marker (0.91, against 0.87 for procalcitonin above 0.04 μg/L), the procalcitonin threshold was derived on the same data, enrolment was confined to patients already selected for surgery, and the authors concluded that procalcitonin should remain ancillary to calcitonin. A design of this kind therefore exists; what is missing is external validation in an unselected population and on other platforms.

Furthermore, procalcitonin is not the only candidate among the alternatives, and the comparison is instructive even though the relevant synthesis fell outside our eligibility criteria. A comparative bivariate meta-analysis of pro-gastrin-releasing peptide published in 2025 pooled eight studies and 4080 patients and evaluated calcitonin within the same populations in the four studies and 1064 patients that allowed it [29]. Two features of that analysis bear directly on procalcitonin. The first is a discordance analysis of the kind no procalcitonin synthesis has performed. Among 356 patients without MTC, 107 had discordant marker results, and for 102 of those, 95%, were calcitonin-positive with a negative second marker; in 29% of the entire non-MTC group, in other words, calcitonin was raised, and the alternative was not. Among 164 patients with MTC, 26 were discordant, 23 of them calcitonin-positive with a negative second marker, and 19 of those 23 had tumours below 10 mm. The second is metrological, and here the comparison favours procalcitonin. Pro-gastrin-releasing peptide has no comparable standardisation, for the reasons set out below [29]. Procalcitonin is in a better position, since its commercial assays descend from a common licensed antibody lineage and agree to within 90% to 99% at the concentrations that matter [21,69]. The marker with the weaker standardisation position is the one whose comparative accuracy has now been formally synthesised against calcitonin; the marker with the stronger one has not been compared with it at all. No study has evaluated procalcitonin and pro-gastrin-releasing peptide head-to-head, a gap the authors of that meta-analysis identify themselves.

Finally, it should be noted that the clinical use and performance of calcitonin and, to a lesser extent, procalcitonin depend on pre-analytical and analytical issues. First, calcitonin requires prompt separation and chilled handling while procalcitonin is stable across ordinary variation in temperature, storage and anticoagulation [22]. Spurious hypercalcitoninaemia may arise from heterophile antibody interference and measuring procalcitonin resolves it [11]. From an analytical point of view it should be underscored that calcitonin is measured by assays whose immunoreactive targets differ, producing results that are poorly comparable between methods and between laboratories [24]. Ten fully automated commercial procalcitonin assay methods delivered acceptable correlation across 176 routine plasma samples [69]. However, differences in assay calibration, antibody specificity, epitopes or recognition, analytical design, and traceability can generate clinically relevant between-method biases, making it technically inappropriate to extrapolate a numerical threshold established with one assay directly to another [74,75,76]. Consequently, no universal procalcitonin or calcitonin concentration triggering a specific clinical action can currently be defined. Clinical application of procalcitonin, and of circulating tumour markers generally, should therefore rely on assay-specific, and where necessary laboratory-specific, decision thresholds that have been appropriately validated for the intended clinical setting. This consideration becomes even more critical for longitudinal monitoring, in which changes over time rather than isolated concentrations often provide the clinically relevant information. Serial measurements should ideally be performed in the same laboratory using the same analytical platform and assay throughout follow-up. If a change in method is unavoidable, an appropriate re-baselining procedure should be undertaken, preferably including parallel measurements with the old and new assays whenever feasible. Importantly, kinetic parameters such as slopes, percentage changes, doubling times, or half-lives should not be calculated by combining results obtained with different assays unless analytical comparability has been formally demonstrated. Thus, harmonisation is not simply an analytical refinement but a prerequisite for translating biomarker concentrations and, particularly, biomarker kinetics into reproducible clinical decision-making.

Pre-analytical stability matters more for kinetics than for single measurements. A doubling time is derived from the slope of log-transformed concentrations over time, so analyte lost before measurement, in amounts that vary with how long each tube stood at room temperature before separation [7,8,9,10], enters the calculation as spurious variation and can shorten or lengthen the apparent doubling time. Calcitonin is exposed to exactly this error, whereas procalcitonin concentrations change little with ordinary variation in temperature and storage [22]. A kinetic index based on procalcitonin should therefore be less sensitive to differences in sample handling between visits and between centres, which is an argument for testing procalcitonin doubling time in multicentre follow-up, where handling cannot be fully standardised. The argument is analytical. It has not been shown that procalcitonin doubling time predicts outcome better than calcitonin doubling time, and stability offers no protection against a change in assay between measurements [68].

Notably, procalcitonin assays were developed, calibrated and validated for the detection of bacterial infection, where the decision limits of interest lie around 0.25 and 0.5 μg/L. The MTC-relevant range sits an order of magnitude lower. On a current electrochemiluminescence method the limit of quantification at a coefficient of variation of 20% is 0.06 μg/L [68], which is precisely the lowest threshold pooled in either meta-analysis. Decisions in this indication are therefore being taken at, and not above, the functional sensitivity of the assay, and an assay characterised at 0.5 μg/L cannot be assumed to perform acceptably at 0.06. The one primary study that examined the point found procalcitonin undetectable in all cured patients on the assay with a limit of quantification of 0.06 μg/L but detectable in 23 and 13 cured patients respectively on two assays with limits of 0.01 μg/L, while all cured patients remained below the 97.5th percentile of healthy adults of about 0.05 μg/L [21]. Greater analytical sensitivity, in other words, produced more detectable results in disease-free patients rather than better discrimination. The deficit is not confined to procalcitonin. The 2025 synthesis records that pro-gastrin-releasing peptide assays likewise lack traceability to a common standard, that no certified reference material or reference measurement procedure exists for it, and that results therefore remain method-dependent and require assay-specific cut-offs [29]. Both proposed successors to calcitonin are held back by the same unfinished metrological work, and neither will reach a guideline threshold until it is done. Any laboratory intending to offer procalcitonin for this indication should establish its own functional sensitivity rather than relying on the manufacturer’s claim, and should state on the report which method generated the threshold it quotes. The second difficulty is biological interference of a kind calcitonin does not share. Procalcitonin rises in bacterial infection, in sepsis, after major surgery and in several inflammatory states, through transcription of the CALC-I gene in tissues that do not normally express it [77,78,79,80]. An elevated serum value in a patient under surveillance who has a concurrent infection can be difficult to interpret and attribute to the underlying disease. Whether the converse holds, and procalcitonin remains usable for monitoring infection in a patient with known MTC, has been examined in isolated reports [43].

Admittedly, the primary studies were mostly retrospective, mostly single-centre, and mostly conducted in tertiary referral populations where the prevalence of MTC bears no relation to that in a routine screening population; prevalence in the series contributing to the 2021 meta-analysis ranged from 0.16% to 60.4% [28]. The reported likelihood ratio of 288, with a confidence interval running from 5.6 to 14,929, is too imprecise to be interpreted. Almost all the pooled data derive from sporadic disease, so extrapolation to hereditary MTC and to RET carriers under surveillance is unsupported, and nothing in these syntheses speaks to the paediatric setting [81,82,83,84].

There are certain limitations of this review to be disclosed. Our search was confined to two databases and to reports indexed as systematic reviews or meta-analyses, so a synthesis published outside those indexes may have been missed; the same two databases were searched by the earlier umbrella review of diagnostic tests in this disease, which retrieved 159 records and 23 eligible syntheses on a single search string [30]. Embase, Web of Science and Scopus were not searched, which departs from the usual recommendation to search several databases with complementary coverage. The reference lists of every included synthesis and of the earlier umbrella review were screened to offset this, and one of the seven included syntheses was in fact identified only by citation searching, which illustrates the point. A synthesis indexed only in Embase or Scopus, or published in a regional journal, could still have been missed, although in a literature this small and this closely interlinked such a synthesis would be less likely to go uncited by the others. The corrected covered area was computed from the study lists as published. The characteristics we extracted from full texts differed in one respect from those tabulated in the earlier umbrella review, which recorded the 2015 systematic review as contributing 265 postoperative and 114 preoperative patients [30]; the source reports 265 postoperative patients of whom 114 were cured, and its two preoperative studies enrolled 1693 patients, of whom 1348 had procalcitonin measured [24]. The discrepancy does not affect any conclusion, but it illustrates how quickly extraction errors propagate through a literature in which each synthesis draws on the last. The remaining uncertainties will need to be addressed through new primary data rather than further evidence synthesis, with the question of clinically relevant thresholds being the most immediate priority. This requires a prospective, multicentre cohort including patients with nodular thyroid disease and patients undergoing postoperative surveillance, with procalcitonin measured on at least two commercially available platforms. The study should use a reference standard independent of calcitonin, derive candidate thresholds in a development cohort, and evaluate them in a separate validation cohort, with reporting in accordance with the Standards for Reporting of Diagnostic Accuracy Studies (STARD) [85]. Two further gaps are worth closing. Procalcitonin doubling time should be compared with the established kinetics of calcitonin and carcinoembryonic antigen in a cohort large enough to relate both to outcome rather than to each other, building on the exploratory agreement already demonstrated [68]. Procalcitonin and pro-gastrin-releasing peptide should also be compared with each other and with calcitonin in the same patients, a gap the most recent synthesis identifies explicitly and which the newest primary studies are already positioned to fill, since two of the eight they pooled measured both alternative markers [29]. And the calcitonin-negative phenotype, where the rationale for procalcitonin is strongest, and the data are almost absent, calls for a registry with prospective measurement of both analytes rather than further case series.

**Figure 1:**
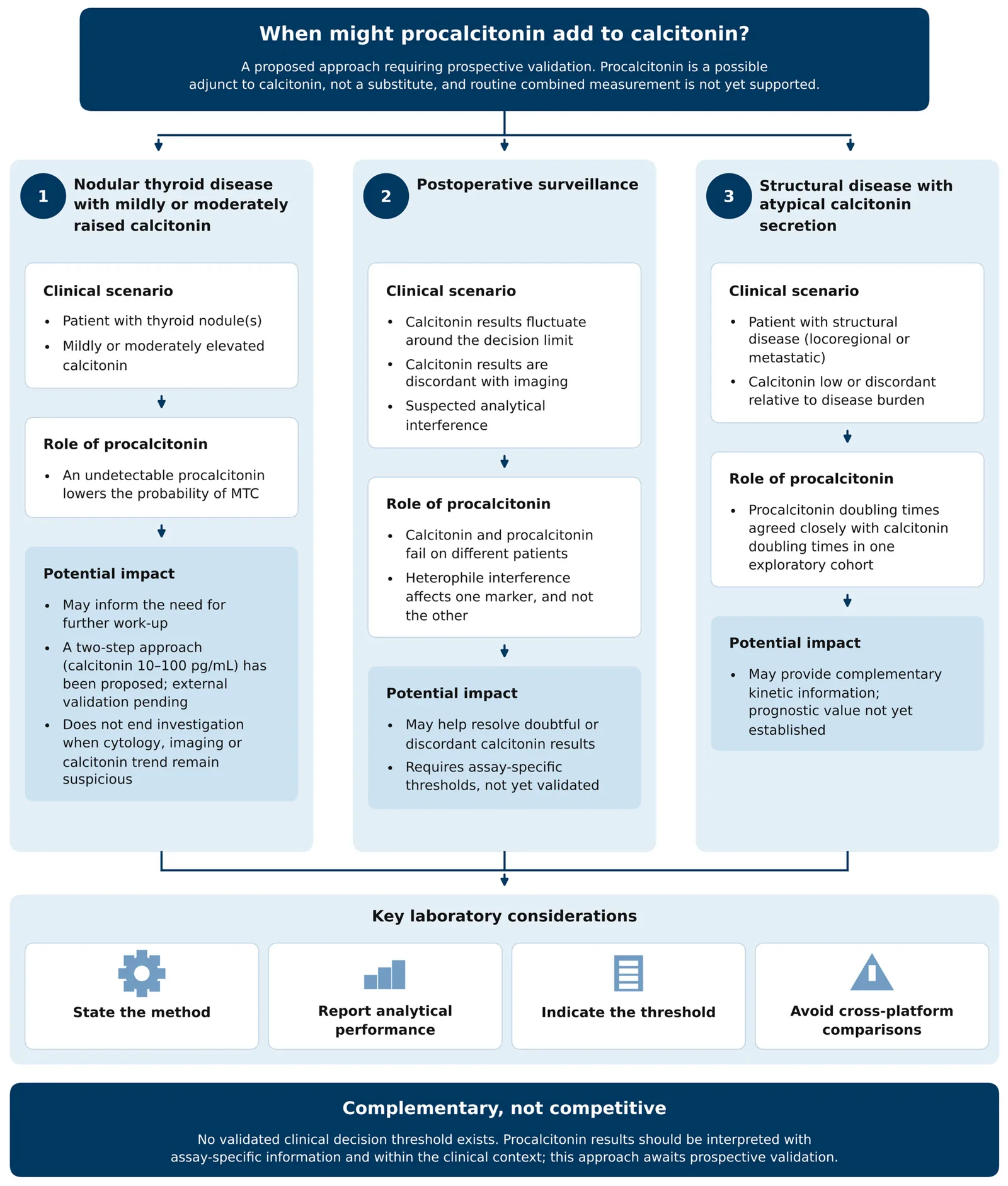
Proposed approach, requiring prospective validation, to measuring procalcitonin together with calcitonin. Three clinical situations in which procalcitonin may add information to calcitonin rather than replace it, with the laboratory information that should accompany every report. MTC, medullary thyroid carcinoma.

On the present evidence, procalcitonin is a possible adjunct to calcitonin rather than a substitute for it, and routine measurement of both is not yet supported. We propose, as an approach that requires prospective validation, three situations in which measuring it together with calcitonin may add information. The first is a patient with nodular thyroid disease and a mildly or moderately raised calcitonin, where an undetectable procalcitonin lowers the probability of MTC but should not by itself end the investigation when cytology, imaging or the calcitonin trend remain suspicious. A two-step approach of this kind, adding procalcitonin when calcitonin lies between 10 and 100 pg/mL, has recently been proposed in a prospective preoperative series and awaits external validation [73]. The second is postoperative surveillance in which calcitonin results fluctuate around the decision limit, are discordant with imaging, or are suspected of analytical interference, since the two markers fail on different patients [54] and heterophile interference affects one, and not the other [11]. The third is a patient with structural disease and atypical calcitonin secretion, where procalcitonin kinetics may add information, although their close agreement with calcitonin kinetics in one exploratory cohort does not establish prognostic value [68]. These situations, offered as a proposal rather than a recommendation, are summarised in Figure 1. Laboratories offering the test should state the method, the functional sensitivity and the threshold applied on every report, and clinicians should not compare values obtained on different platforms without evidence that they are comparable.

## 5. Conclusions

Eleven years of evidence synthesis suggest that procalcitonin can detect MTC before surgery and identify recurrence during follow-up, with accuracy approaching that of calcitonin and more favourable analytical characteristics. However, these eleven years have not yielded a validated clinical decision threshold, have not adequately evaluated the marker in the calcitonin-negative phenotype in which it could be most valuable, and have only recently begun to examine its kinetics as a prognostic biomarker. The published reviews are largely concordant, in part because they draw on overlapping source studies, while the design of the underlying studies does not allow superiority over calcitonin to be demonstrated, even in principle. Procalcitonin may therefore have a role as a complement to calcitonin in diagnosis and surveillance, but its routine use alongside calcitonin awaits validated, method-specific thresholds. Further advancement will require prospective, multiplatform studies establishing clinically relevant thresholds against an independent reference standard.

## Figures and Tables

**Table 1:** Characteristics of the seven evidence syntheses included in the umbrella review.

Evidence Synthesis	Design	Search To	Primary Reports/Patients	Clinical Setting	Findings Concerning Procalcitonin
Trimboli and Giovanella, 2015, *Clin Chem Lab Med* [25]	SR without MA	31 Dec 2014	11 reports/18 patients	Calcitonin-negative MTC	Procalcitonin was measured in one patient in the whole series, falling from 0.21 μg/L before surgery to below 0.1 μg/L afterward. CEA was measured in 11 cases and was normal in 10. The authors set out the mechanistic condition on which procalcitonin could help: aberrant processing of mature calcitonin would leave procalcitonin raised, whereas a defect of the secretory apparatus would leave it low as well.
Trimboli et al., 2015, *Endocr Relat Cancer* [24]	SR without MA	18 Feb 2015	Nine of 39 records/265 postoperative patients (114 cured, 151 recurrent or persistent) plus 1693 preoperative patients in two studies, of whom 1348 had procalcitonin measured	Preoperative diagnosis and postoperative follow-up	First synthesis devoted to procalcitonin in MTC. Per-study sensitivity in recurrent disease was 100% in four series, 91.2% in the largest and 84.0% in one; specificity in cured patients ranged from 57.1% to 100%. Thresholds of 0.1, 0.15, 0.25 and 0.5 μg/L were in use. Concentrations up to 0.28 μg/L were recorded in athyreotic and healthy subjects.
Karagiannis et al., 2016, *Anticancer Res* [26]	SR without MA	March 2016	15 of 184 records, three of them case reports/485 MTC patients and 1035 comparators	Preoperative diagnosis and postoperative follow-up	Procalcitonin was judged useful alongside calcitonin rather than in its place. The authors noted that no threshold could be recommended on the available data, while observing that most studies had adopted 0.1 μg/L. Eligibility was restricted to English, French and Greek.
Trimboli and Giovanella, 2018, *Endocrinol Metab* (Seoul) [27]	SR with MA, univariate random effects	May 2018	Eight reports in the review, five pooled/296 patients (140 recurrent, 108 with no evidence of disease and 48 newly diagnosed or biochemically stable)	Postoperative surveillance	Pooled sensitivity for recurrent disease 96% (95% CI 92–99), I-squared 0% (95% CI 0–64.1), Egger test 3.16 (*p* = 0.99). Pooled specificity 96% (95% CI 87–100) from three studies only, I-squared 66.6%. Thresholds 0.06–0.32 μg/L, two of them derived by ROC analysis on the same data. QUADAS-2 applied to all five.
Giovanella et al., 2021, *J Clin Endocrinol Metab* [28]	SR with MA, bivariate and HSROC	May 2021	11 of 242 records/5817 patients, 336 with MTC	Prediction of MTC in nodular thyroid disease and treatment monitoring	Pooled sensitivity 0.90 (95% CI 0.71–0.97), specificity 1.00 (95% CI 0.85–1.00), LR+ 288 (95% CI 5.6–14,929.3), LR- 0.10 (95% CI 0.03–0.33), AUC 0.97 (95% CI 0.95–0.98). Postoperative subgroup (four studies, 364 patients, MTC prevalence 48.6%): sensitivity 0.93 (0.85–0.97), specificity 0.91 (0.20–1.00). Thresholds 0.06–0.50 μg/L. Meta-regression identified assay platform and threshold as significant influences. Registered as PROSPERO CRD42021253278.
Zarkesh et al., 2022, *Pathol Res Pract* [40]	SR without MA	2021; publications before 2000 excluded	76 of 507 records, 11 of them addressing procalcitonin, alongside 37 on calcitonin, 14 on CEA, 6 on chromogranin A, 5 on ProGRP and 3 on CA 19-9	Circulating markers in MTC generally	Reproduces per-study accuracy figures for procalcitonin without pooling: preoperative sensitivity 95.5% (107/112) in the largest series and negative predictive values of 98.1%, 96.3% and 95.4% at 0.05, 0.10 and 0.15 μg/L; postoperative sensitivity 84–100% and specificity 57.1–100%. Calcitonin was retained as the most sensitive marker, procalcitonin credited with potential to replace it in non-secretory disease, and marker combinations held to improve accuracy.
Abaalkhail et al., 2024, *Gland Surg* [41]	SR without MA	9 March 2023	32 studies of 140 screened records, 11 of them added by citation searching/101 patients	Calcitonin-negative MTC	Largest series of the phenotype. Procalcitonin was measured in two patients, raised before surgery and normal afterwards in one and normal throughout in the other; in a further patient it identified relapse when all other neuroendocrine markers were negative. CEA was measured in 59 patients and was normal in 51. Calcitonin immunohistochemistry was positive in 57 of 93 tumours. The authors state the same mechanistic conditional as the 2015 review, reached independently. Registered as PROSPERO CRD42023413564 on 12 April 2023, after the search had closed; no risk-of-bias assessment was performed.

CEA, carcinoembryonic antigen; CA 19-9, carbohydrate antigen 19-9; CI, confidence interval; HSROC, hierarchical summary receiver operating characteristic; LR+, positive likelihood ratio; LR-, negative likelihood ratio; MA, meta-analysis; MTC, medullary thyroid carcinoma; ProGRP, pro-gastrin-releasing peptide; QUADAS-2, Quality Assessment of Diagnostic Accuracy Studies, version 2; ROC, receiver operating characteristic; SR, systematic review. Reference [40] describes itself as a systematic review and reports a reproducible three-database search with explicit eligibility criteria and a flow diagram, and is treated as eligible on that basis, although the journal classifies it as a review article.

**Table 2:** AMSTAR-2 appraisal of the included evidence syntheses, restricted to the seven critical domains.

Evidence Synthesis	Item 2 Protocol	Item 4 Search	Item 7 Excluded Studies	Item 9 Risk of Bias	Item 11 Statistical Methods	Item 13 Bias in Interpretation	Item 15 Publication Bias	Overall Confidence
Trimboli and Giovanella, 2015 [25]	No	Partial yes	No	No	NA	No	NA	Critically low
Trimboli et al., 2015 [24]	No	Partial yes	No	No	NA	No	NA	Critically low
Karagiannis et al., 2016 [26]	No	No	No	No	NA	No	NA	Critically low
Trimboli and Giovanella, 2018 [27]	No	Partial yes	Yes	Yes	No	Yes	No	Critically low
Giovanella et al., 2021 [28]	Yes	Yes	No	Yes	Yes	Yes	Yes	Low
Zarkesh et al., 2022 [40]	No	No	No	No	NA	No	NA	Critically low
Abaalkhail et al., 2024 [41]	No	No	No	No	NA	No	NA	Critically low

Item 2, protocol registered before commencement of the review; item 4, adequacy of the literature search; item 7, list of excluded studies with justification; item 9, satisfactory technique for assessing risk of bias in primary studies; item 11, appropriateness of the methods used for statistical combination; item 13, risk of bias accounted for when interpreting results; item 15, investigation of publication bias. NA, not applicable because no meta-analysis was performed. Partial yes is available in AMSTAR-2 only for items 2, 4, 7, 8 and 9. For accuracy reviews, item 11 was rated yes only for hierarchical (bivariate or HSROC) models and item 15 only for a test appropriate to accuracy data.

**Table 3:** Citation matrix of the primary reports on procalcitonin included in five of the seven syntheses.

Primary Report	Trimboli 2015 [24]	Karagiannis 2016 [26]	Trimboli 2018 [27]	Giovanella 2021 [28]	Zarkesh 2022 [40]
Bihan et al., 2003 [52]	X	X	X		
Bolko et al., 2003 [53]	X	X	X		X
Algeciras-Schimnich et al., 2009 [54]	X	X	X	X	X
Giovanella et al., 2010 [55]		X		X	
Azevedo et al., 2010 [50]		X		X	
Kowalska et al., 2010 [51]		X			
Kaczka et al., 2010 [43]	X		X		X
Walter et al., 2010 [56]	X	X	X	X	X
Canale et al., 2011 [47]		X			
Kratzsch et al., 2011 [57]	X	X	X		X
Kaczka et al., 2012 [44]	X	X	X	X	X
Giovanella et al., 2013 [58]	X	X		X	X
Lim et al., 2014 [45]		X			
Machens et al., 2014 [59]	X	X			X
Bugalho et al., 2014 [48]		X			
Brutsaert et al., 2015 [49]		X			
Lim et al., 2016 [46]				X	X
Trimboli et al., 2018 [60]			X	X	X
Giovanella et al., 2018 [61]				X	
Giovanella et al., 2021 [62]				X	
Kratzsch et al., 2021 [21]				X	
Total reports included	9	15	8	11	10

N = 53 filled cells, r = 21 distinct primary reports, c = five syntheses; corrected covered area 38.1%, and 44.4% after removal of 12 structural zeros. The count for reference [27] is the eight reports retained in its systematic review, five of which were carried into its meta-analysis. The count for reference [40] is the ten distinct primary reports tabulated in that paper under procalcitonin; its flow diagram states eleven, the difference arising because one report is listed twice under two clinical indications. The two included syntheses of calcitonin-negative disease [25,41] are not represented as columns: they searched for MTC without calcitonin secretion rather than for procalcitonin. Of the three primary reports in them that carry a procalcitonin value, one already appears as a row [49]; the other two, neither included by any procalcitonin-specific synthesis, are a case report of relapse detected by procalcitonin [63] and a case series [64], and so have no column to occupy. Nor is the excluded pro-gastrin-releasing peptide meta-analysis [29], whose primary studies were selected for a different index test.

**Table 4:** Summary of evidence statements with the syntheses supporting them and the certainty attached.

Statement	Supporting Syntheses	Certainty
During postoperative surveillance, procalcitonin identifies persistent or recurrent MTC with pooled sensitivity and specificity of about 96%.	[27], and the postoperative subgroup of [28]	Low (risk of bias; inconsistency and imprecision for specificity)
In pooled diagnostic and monitoring populations, four of the eleven studies being exclusively postoperative, procalcitonin discriminated MTC from non-MTC with pooled sensitivity of 0.90 and specificity approaching unity, at an area under the curve of 0.97.	[28]	Low (risk of bias, indirectness)
No decision threshold has been validated. Values between 0.06 and 0.50 μg/L have been applied, several derived post hoc from the data on which they were then tested, and threshold and assay platform are demonstrable determinants of the pooled estimates.	[24,26,27,28]	High (consistent across every synthesis addressing it)
Procalcitonin does not resolve the problem of calcitonin-negative MTC. Across 101 published patients with that phenotype, in three syntheses, the marker was measured in three.	[25,41], and the excluded [42]	Very low (case-level data)
Measuring procalcitonin together with calcitonin performed better than either alone during follow-up in one cohort.	Single-cohort data reported within [27]	Very low (single retrospective cohort, imprecision)
Procalcitonin doubling times agree closely with calcitonin doubling times; their prognostic value has not been established.	Not addressed by any included synthesis; first direct evidence published in 2026 [68]	Very low (single small exploratory study, imprecision; not addressed by any synthesis)
The analytical behaviour of procalcitonin, and the common provenance of commercial assays, favour it over calcitonin for sample handling and inter-method comparability.	Asserted as rationale in [24,27,28]; supported by primary method-comparison data [21,69]	Moderate for the analytical claim, untested as a clinical outcome

Certainty was judged with the GRADE approach for test accuracy and refers to the body of evidence behind each statement, not to the AMSTAR-2 confidence in individual reviews (Table 2); reasons for rating down are given in parentheses. CI, confidence interval; MTC, medullary thyroid carcinoma.

## Data Availability

No new data were created.

## Supplementary Materials

The following supporting information can be downloaded at: https://www.mdpi.com/article/10.3390/jcm15218255/s1, Table S1: search strategies for PubMed/MEDLINE and the Cochrane Library, with the citation-searching arm and the records retrieved from each source. Figure S1: PRISMA 2020 flow diagram of identification, screening and inclusion, with evidence syntheses rather than primary studies as the units of synthesis. File S1: Completed PRISMA 2020 checklist.
