# Supplementary material for: Procalcitonin in Medullary Thyroid Carcinoma: An Umbrella Review of Systematic Reviews and Meta-Analyses
Source: J Clin Med. 2026 Oct 23;15(21):8255. doi: 10.3390/jcm15218255 (PMC13764064; doi:10.3390/jcm15218255)
Supplement: Supplementary file 1 [file jcm-15-08255-s001.zip › PRISMA_2020_checklist.pdf]

# PRISMA 2020 checklist

## Procalcitonin in medullary thyroid carcinoma: an umbrella review of systematic reviews and meta-analyses

*This umbrella review takes systematic reviews and meta-analyses, rather than primary studies, as its units of synthesis. PRISMA 2020 items are therefore read at the level of the evidence syntheses included.*

| Item | Section / topic | Checklist item | Location where item is reported |
|---|---|---|---|
| **TITLE** | | | |
| 1 | Title | Identify the report as a systematic review. | Title page |
| **ABSTRACT** | | | |
| 2 | Abstract | See the PRISMA 2020 for Abstracts checklist. | Abstract |
| **INTRODUCTION** | | | |
| 3 | Rationale | Describe the rationale for the review in the context of existing knowledge. | Introduction, paras 1–3 |
| 4 | Objectives | Provide an explicit statement of the objective(s) or question(s) the review addresses. | Introduction, final para |
| **METHODS** | | | |
| 5 | Eligibility criteria | Specify the inclusion and exclusion criteria for the review and how studies were grouped for the syntheses. | Methods, Eligibility criteria |
| 6 | Information sources | Specify all databases, registers, websites, organisations, reference lists and other sources searched or consulted, and the date when each was last searched or consulted. | Methods, Search and study selection |
| 7 | Search strategy | Present the full search strategies for all databases, registers and websites, including any filters and limits used. | Methods, Search and study selection; full strategies in Supplementary Table S1 |
| 8 | Selection process | Specify the methods used to decide whether a study met the inclusion criteria, including how many reviewers screened each record and each report, whether they worked independently, and if applicable, details of automation tools used. | Methods, Search and study selection |
| 9 | Data collection process | Specify the methods used to collect data from reports, including how many reviewers collected data from each report, whether they worked independently, any processes for obtaining or confirming data from investigators, and if applicable, details of automation tools used. | Methods, Search and study selection (final paragraph) |
| 10a | Data items (outcomes) | List and define all outcomes for which data were sought. | Methods, Search and study selection (final paragraph); Methods, Synthesis |
| 10b | Data items (other variables) | List and define all other variables for which data were sought. | Methods, Search and study selection (final paragraph) |
| 11 | Study risk of bias assessment | Specify the methods used to assess risk of bias in the included studies, including details of the tool(s) used, how many reviewers assessed each study and whether they worked independently. | Methods, Methodological quality (AMSTAR-2) |
| 12 | Effect measures | Specify for each outcome the effect measure(s) used in the synthesis or presentation of results. | Methods, Synthesis |
| 13a | Synthesis methods (eligibility) | Describe the processes used to decide which studies were eligible for each synthesis. | Methods, Synthesis; Methods, Overlap of primary studies |
| 13b | Synthesis methods (preparation) | Describe any methods required to prepare the data for presentation or synthesis. | Methods, Search and study selection (final paragraph) |
| 13c | Synthesis methods (tabulation) | Describe any methods used to tabulate or visually display results of individual studies and syntheses. | Methods, Overlap of primary studies; Tables 1-4 |

| Item | Section / topic | Checklist item | Location where item is reported |
|---|---|---|---|
| 13d | Synthesis methods (results) | Describe any methods used to synthesise results and provide a rationale for the choice(s); if meta-analysis was performed, describe the model(s), method(s) to identify heterogeneity, and software package(s) used. | Methods, Synthesis (narrative synthesis; no pooling across syntheses, with rationale) |
| 13e | Synthesis methods (heterogeneity) | Describe any methods used to explore possible causes of heterogeneity among study results. | Methods, Overlap of primary studies (corrected covered area) |
| 13f | Synthesis methods (robustness) | Describe any sensitivity analyses conducted to assess robustness of the synthesised results. | Methods, Overlap of primary studies (structural zeros; pairwise CCA) |
| 14 | Reporting bias assessment | Describe any methods used to assess risk of bias due to missing results in a synthesis. | Methods, Methodological quality (AMSTAR-2 item 15) |
| 15 | Certainty assessment | Describe any methods used to assess certainty (or confidence) in the body of evidence for an outcome. | Methods, Certainty of evidence (GRADE approach for test accuracy); Table 4 |
| **RESULTS** | | | |
| 16a | Study selection | Describe the results of the search and selection process, from the number of records identified in the search to the number of studies included in the review, ideally using a flow diagram. | Results, Included evidence syntheses; PRISMA flow diagram (Figure S1) |
| 16b | Study selection (exclusions) | Cite studies that might appear to meet the inclusion criteria, but which were excluded, and explain why they were excluded. | Results, Included evidence syntheses (two named full-text exclusions with reasons) |
| 17 | Study characteristics | Cite each included study and present its characteristics. | Table 1; Results, Included evidence syntheses |
| 18 | Risk of bias in studies | Present assessments of risk of bias for each included study. | Table 2; Results, Methodological quality |
| 19 | Results of individual studies | For all outcomes, present for each study summary statistics for each group and an effect estimate and its precision, ideally using structured tables or plots. | Table 1; Results, preoperative and postoperative sections |
| 20a | Results of syntheses | For each synthesis, briefly summarise the characteristics and risk of bias among contributing studies. | Results, Methodological quality; Results, Overlap of the primary evidence base |
| 20b | Results of syntheses (estimates) | Present results of all statistical syntheses conducted; if meta-analysis was done, present summary estimates and their precision and measures of statistical heterogeneity. | Results, preoperative and postoperative sections |
| 20c | Results of syntheses (heterogeneity) | Present results of all investigations of possible causes of heterogeneity among study results. | Results, Overlap of the primary evidence base; Results, Discordant extraction; Results, Decision thresholds |
| 20d | Results of syntheses (robustness) | Present results of all sensitivity analyses conducted to assess the robustness of the synthesised results. | Results, Overlap of the primary evidence base (adjusted and pairwise CCA); Table 3 |
| 21 | Reporting biases | Present assessments of risk of bias due to missing results for each synthesis assessed. | Table 2 (AMSTAR-2 item 15); Results, Methodological quality |
| 22 | Certainty of evidence | Present assessments of certainty (or confidence) in the body of evidence for each outcome assessed. | Table 4 |
| **DISCUSSION** | | | |
| 23a | Discussion (interpretation) | Provide a general interpretation of the results in the context of other evidence. | Discussion, opening paragraphs |
| 23b | Discussion (limitations of evidence) | Discuss any limitations of the evidence included in the review. | Discussion, paragraphs on the reference standard, the design of the primary |

| Item | Section / topic | Checklist item | Location where item is reported |
|---|---|---|---|
| | | | series and analytical considerations |
| 23c | Discussion (limitations of review) | Discuss any limitations of the review processes used. | Discussion, paragraph on the limitations of this review |
| 23d | Discussion (implications) | Discuss implications of the results for practice, policy and future research. | Discussion, final paragraphs; Figure 1; Conclusions |
| **OTHER INFORMATION** | | | |
| 24a | Registration and protocol | Provide registration information for the review, including register name and registration number, or state that the review was not registered. | Methods, Design, protocol and reporting. Registered retrospectively at the Open Science Framework, DOI 10.17605/OSF.IO/B97FK |
| 24b | Protocol access | Indicate where the review protocol can be accessed, or state that a protocol was not prepared. | Protocol deposited at the Open Science Framework, DOI 10.17605/OSF.IO/B97FK |
| 24c | Amendments | Describe and explain any amendments to information provided at registration or in the protocol. | Methods, Certainty of evidence: GRADE approach for test accuracy, and Deeks' test under AMSTAR-2 item 15, adopted during peer review as amendments to the registered protocol |
| 25 | Support | Describe sources of financial or non-financial support for the review, and the role of the funders or sponsors in the review. | Back matter, Funding |
| 26 | Competing interests | Declare any competing interests of review authors. | Back matter, Conflicts of Interest |
| 27 | Availability of data, code and other materials | Report which of the following are publicly available and where they can be found: template data collection forms; data extracted from included studies; data used for all analyses; analytic code; any other materials used in the review. | Back matter, Data Availability Statement |

*Reference: Page MJ, McKenzie JE, Bossuyt PM, Boutron I, Hoffmann TC, Mulrow CD, et al. The PRISMA 2020 statement: an updated guideline for reporting systematic reviews. BMJ 2021;372:n71.*
