# Supplementary material for: Procalcitonin in Medullary Thyroid Carcinoma: An Umbrella Review of Systematic Reviews and Meta-Analyses
Source: J Clin Med. 2026 Oct 23;15(21):8255. doi: 10.3390/jcm15218255 (PMC13764064; doi:10.3390/jcm15218255)
Supplement: Supplementary file 1 [file jcm-15-08255-s001.zip › Table_S1_search_strategies.pdf]

Supplementary Table S1. Search strategies

Procalcitonin in medullary thyroid carcinoma: an umbrella review of systematic reviews and meta-analyses

Source	Search strategy as run	Records retrieved
PubMed/MEDLINE	Query as entered:<br>#1 (systematic review OR meta-analysis) AND procalcitonin AND medullary thyroid cancer<br><br>Query as translated by PubMed automatic term mapping:<br>("systematic review"[Publication Type] OR "systematic reviews as topic"[MeSH Terms] OR "systematic review"[All Fields] OR ("meta analysis"[Publication Type] OR "meta analysis as topic"[MeSH Terms] OR "meta analysis"[All Fields])) AND ("procalcitonin"[Supplementary Concept] OR "procalcitonin"[All Fields] OR "procalcitonin"[MeSH Terms] OR "procalcitonine"[All Fields]) AND ("thyroid cancer medullary"[Supplementary Concept] OR "thyroid cancer medullary"[All Fields] OR "medullary thyroid cancer"[All Fields])<br><br>No language restriction and no additional filters were applied. Results sorted by most recent. The search was run on 30 June 2026, which defines the end of the search period, so no separate date filter was required.	8
Cochrane Library	#1 "procalcitonin" [Title Abstract Keyword]<br>#2 "medullary thyroid" [Title Abstract Keyword]<br>#3 #1 AND #2<br><br>Limited to Cochrane Reviews, Cochrane Library publication date to June 2026. Word variations searched. Cochrane Database of Systematic Reviews, Issue 9 of 12, September 2026.	0
Citation searching	Reference lists of all retrieved reports and of the 2023 umbrella review of diagnostic tests in medullary thyroid carcinoma were screened for evidence syntheses not identified by the database searches.	1
**Total**		**9**

Searches were run from database inception to 30 June 2026, with no language restriction. The units sought were systematic reviews and meta-analyses rather than primary studies.

Both database searches and the citation search are reported in full; no entries remain outstanding.
